# A biophysical model for transcription factories

**DOI:** 10.1186/2046-1682-6-2

**Published:** 2013-02-09

**Authors:** Ana Z Canals-Hamann, Ricardo Pires das Neves, Joyce E Reittie, Carlos Iñiguez, Shamit Soneji, Tariq Enver, Veronica J Buckle, Francisco J Iborra

**Affiliations:** 1 MRC Molecular Haematology Unit, Weatherall Institute of Molecular Medicine, John Radcliffe Hospital, Headington, Oxford, OX3 9DS, UK; 2 Departamento de Biotecnología, Universidad de Alicante, Alicante, 03080, Spain; 3 Departamento de Biología Molecular y Celular, Centro Nacional de Biotecnología, CSIC, Darwin 3, Campus de Canto Blanco, Madrid, 28049, Spain

**Keywords:** Epigenetics, Biophysics, H4K16Ac, BrUTP, Transcription Factories, RNA pol II, Nuclear organization

## Abstract

**Summary:**

Transcription factories are nuclear domains where gene transcription takes place although the molecular basis for their formation and maintenance are unknown. In this study, we explored how the properties of chromatin as a polymer may contribute to the structure of transcription factories. We found that transcriptional active chromatin contains modifications like histone H4 acetylated at Lysine 16 (H4K16ac). Single fibre analysis showed that this modification spans the entire body of the gene. Furthermore, H4K16ac genes cluster in regions up to 500 Kb alternating active and inactive chromatin. The introduction of H4K16ac in chromatin induces stiffness in the chromatin fibre. The result of this change in flexibility is that chromatin could behave like a multi-block copolymer with repetitions of stiff-flexible (active-inactive chromatin) components. Copolymers with such structure self-organize through spontaneous phase separation into microdomains. Consistent with such model H4K16ac chromatin form foci that associates with nascent transcripts. We propose that transcription factories are the result of the spontaneous concentration of H4K16ac chromatin that are in proximity, mainly in cis.

## Background

Transcription in eukaryotes is organized in transcription factories (TFs), which are nuclear domains where several genes are grouped to be transcribed together [1,2][3-5]. The current opinion is that the genes in a TF interact by a looping mechanism [5-8]. It has been suggested that chromatin looping plays an important role in controlling gene activity by bringing together promoters and enhancers or TFs [9]. Some studies suggest that promoter-enhancer loops are maintained by the interaction of proteins associated with these cis-regulatory elements [10]. This interaction precedes chromatin activation, which is required for gene relocation to the TF by an unknown mechanism [11]. It has been proposed that TFs are maintained by depletion attraction forces (excluding volume effect) between RNA pol II molecules [12]. However, experimental evidence has shown that genes remain at the factory even when active RNA pol II is not present [13]. This makes the excluding volume model very improbable and suggests that this structure is not the result of transcription. Instead experimental evidence points to histone acetylation as being responsible for loop formation [11]. For these reasons we explored the possible contribution of chromatin acetylation in the formation of TFs.

Chromatin at the TF is decondensed [2] and contains active transcription marks like histone acetylation or H3K36me3 [14]. Among all the possible Lysine residues that can be acetylated, H4K16Ac is very special because it prevents the formation of compacted chromatin by inhibiting the inter-fibre interaction [15-18]. Moreover, H4K16 acetylation is associated with both active chromatin [19] and with the active transcription marker H3K4me3 [19-21].

## Results and discussion

**Figure 1:**
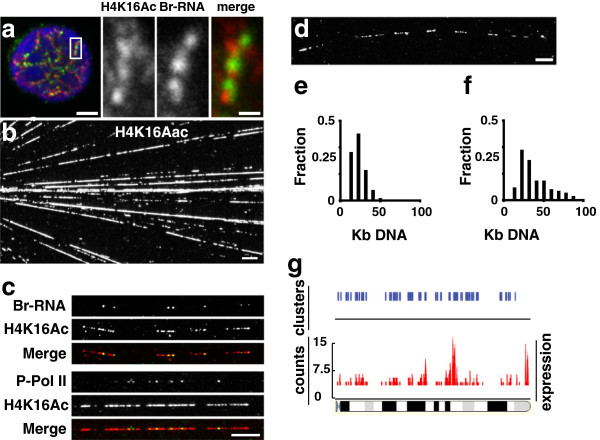
**Transcription on acetylated chromatin.** (**a**) The nascent Br-RNA (green) and H4 K16Ac (red) signals are closely associated. BrUTP incorporation in human lymphocyte was carried out for 15 min and after fixation immunolabeled together with histone H4 K16Ac (rabbit antibody). (**b**) The deconstruction of cell nuclei. After sarkosyl treatment, chromatin was spread and immunolabelled with H4 K16ac, to show tracks of hyperacetylated chromatin. (**c**) The colocalisation of Br-RNA after BrUTP and H4 K16ac. Br-RNA appears as little spots on tracks of acetylated chromatin, equivalent images were obtained when P-RNA pol II (Ser^2^) antibody (H5) was used. (**d**) Tracks of acetylated chromatin appeared in clusters. (**e**) The distribution of sizes of chromatin acetylated tracks. (**f**) The distribution of sizes of chromatin between consecutive acetylated tracks. (**g**) Expression data from FCDP mix cells on mouse chromosome 10. Expressed genes tend to cluster along the chromosome. For cluster analysis we used a 500 Kb window. When clustering was significant (p>0.95) a blue line is drawn. Bars: a = 2 μm, merge = 200 nm; b, c, d = 10 μm.

To confirm whether H4K16Ac is associated with active chromatin, we analysed the distribution of H4K16Ac in the nucleus of the TFs of circulating lymphocytes. The TFs were visualised as sites of incorporation of Br-UTP into nascent RNA. TFs appeared as discrete foci distributed along the edge of condensed chromatin (Figure 1a) as previously described in other cell types [2,22,23]. H4K16Ac was scattered in foci overlapping or very close to these Br-RNA sites (Figure 1a). To study the extent and degree of the hyper-acetylated chromatin in individual transcription units (TUs), we deconstructed the nuclei of these cells by making chromatin spreads. Under these conditions active RNA polymerases and epigenetic modifications of the chromatin are preserved. This treatment disassembled nuclei and spread templates over a wide area. The DNA adopts a linear structure with no visible nucleosomes [22] and 95% of active polymerases remain associated with the DNA [24]. When we stained the chromatin spreads with antibodies against H4K16Ac, they showed almost continuous fluorescent tracks along the DNA fibres (Figure 1b). These H4K16Ac tracks corresponded with active chromatin, as demonstrated by co-localisation of H4K16Ac with the nascent transcripts that were labelled either *in vivo* or *in vitro* by using Bromo-Uridine (BrU) or BrUTP respectively. The area covered by acetylated histones was larger than that stained by the nascent transcripts (Figure 1c). This was to be expected because histone acetylation extends over long stretches of genes, whilst only a few RNA pol II molecules are ever found on a given gene [22]. Nevertheless, to demonstrate that the distribution of active RNA pol II molecules is not an artifact of non-natural nucleotide incorporation, we carried out chromatin spreads with cells where transcription was not labelled with BrU. Our experiments demonstrated a similar co-localisation of H4K16Ac with P-RNA pol II (hyper-phosphorylated Ser_2_) (Figure 1c). 

The chromatin spreading technique allowed us to measure the length of H4K16Ac tracks. The distribution of H4K16Ac stretches showed a lognormal distribution with average size of ~15 Kb (Figure 1e). H4K16Ac tracks rarely appeared isolated, instead they tended to cluster, spanning several hundreds of Kb (348 ± 90; range 235–530 Kb) (Figure 1d). The extension of the gaps between two consecutive H4K16Ac tracks in the cluster showed a lognormal distribution with an average distance of ~30 Kb (Figure 1f). The analysis of the polymerases loaded onto H4K16Ac tracks showed that not all the tracks were stained with Br-RNA or P-RNA pol II. The number of nascent transcripts or P-RNA pol II per track was low (0.7 ± 1 transcripts/track and 0.8 ± 0.9 P-RNA pol II/track). This was in accordance with our previous findings, suggesting that most of the TUs contain one molecule of RNA pol II [22]. The fact that some H4K16Ac tracks of chromatin were not associated to RNA pol II or Br-RNA could reflect a temporal discrepancy between the transcription and acetylation processes of chromatin. Indeed, transcription by RNA pol II takes only a few minutes [25-27] while deacetylation of active chromatin can take several hours [28], providing a molecular memory of recently-transcribed chromatin. On the other hand, H4K16Ac tracks are not a special feature of lymphocytes as we were able to find the same chromatin organisation in all the mammalian cell types tested including: Hela, Epstein Barr transformed lymphocytes, human lymphocytes, primary human fibroblasts, primary mouse fibroblasts and murine erythroleukemia cells (both differentiated and undifferentiated).

The clusters in all the different cell types analysed were identical with respect to the number of TUs (8 ± 2 TUs/Cluster), suggesting that co-linear active genes expressed at the same time, in agreement with the analysis of expression data using FDCP mix cells [29]. The sliding window analysis (applying a window of 500 Kb) over the entire genome showed that genes are active in clusters (Figure 1g), in accordance with our chromatin spreads data. Moreover, our results are consistent with the co-expression data after a Serial Analysis of Gene Expression where the cluster size was <500 Kb [30]. From these data we can conclude that co-linear TUs are active at the same time in the same cell.

### How are these TUs organised in the cell nucleus?

**Figure 2:**
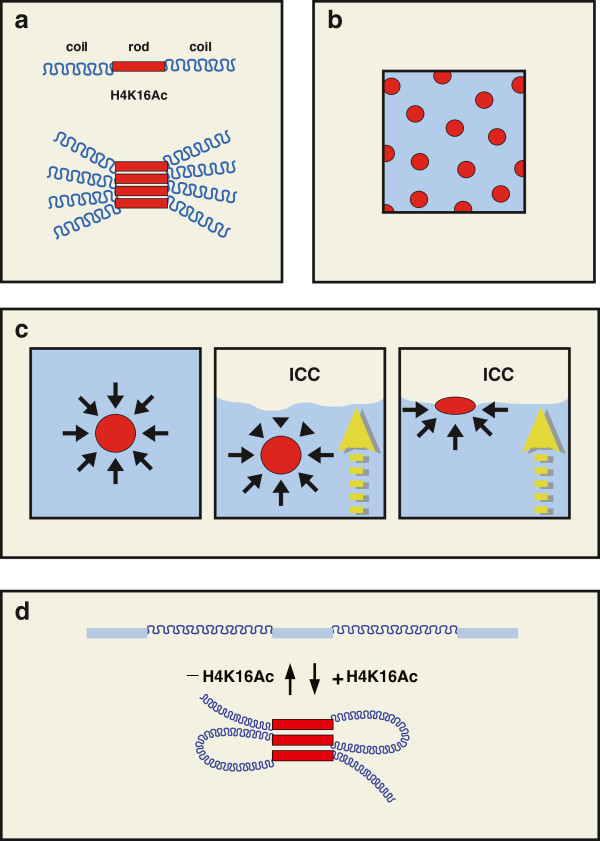
**Multi-block copolymer.** (**a**) Chromatin exists as a multi-block copolymer with flexible and stiff (coil-rod) chromatin. Stiff blocks self-interact creating an anisotropic crystalline phase (pile of red blocks). (**b**) These segregated blocks generate a symmetric microphase pattern where the stiff phase (minor component) self organise in micro-spheres (red circles) embedded in the major phase of flexible DNA (blue background). (**c**) The organization of the active chromatin microphase in the cell nucleus. Microspheres remain in a fixed position due to the balance of opposing forces. In the cell nucleus the existence of interphases creates an imbalance between the forces acting in opposing directions, with a net force pushing the microspheres to the interphase. This explains the position of active chromatin at the edge of the condensed chromatin. (**d**) Model of self-organization of active chromatin. Collinear active gene chromatin is hyper-acetylated which results in its self-interaction with neighbouring TUs. These interactions are destroyed by the deacetylation of chromatin.

Collinear active TUs are enriched in H4K16Ac which confers stiffness and inhibits inter-fiber interaction [15-17]. In this way, chromatin appears as a multi-block copolymer with stiff and flexible monomers (rod-coil)_n_ system, where the rod is the stiff active TU. The multi-block copolymers function as amphiphiles whose components segregate into domains to avoid unfavourable contact with each other. In these systems, complete phase separation is prevented by the covalent linkage between the components [31]. The rod block does not have the same conformational entropy as the coil block and this restricts homogeneous packaging. In consequence anisotropic interactions occur between the stiff blocks ending in a liquid crystalline domain where the different TUs are aligned in a high order smectic phase [31] (Figure 2a). Multi-block copolymers can adopt many different structures depending on the relative proportions of the rod and coil phases. For example, when the rod phase is lower than 20% the structures obtained are microspheres [32] (Figure 2b). A calculation of the amount of active chromatin in a Hela cell line gives a proportion of rods to coils of ~12%, which is consistent with active chromatin separated in many microspheres. These spheres for H4K16Ac chromatin were observed in the cell nucleus of human lymphocytes (the shape factor was 0.93 + 0.05). Microspheres are regularly distributed in artificial polymers with regular coils and rods resulting from the repulsion forces of coils pushing in all directions. In the cell nucleus TFs are not regularly distributed because the sizes of genes and intergenic distances are not as regular as in artificial polymers. Moreover, H4K16Ac foci concentrated at the edge of condensed chromatin (Figure 1a). A possible reason for this discrepancy is that chromatin cohabits with the inter chromatin compartment (ICC), which is composed by RNPs and proteins. This results in a biphasic system where the ICC (inelastic phase) segregates from chromatin [33], creating an interphase between both components. Under these conditions microspheres containing H4K16Ac may be pushed by the coil polymers to the interphase between chromatin and ICC (Figure 2c), resulting in the localisation along the edge of the chromatin as observed. 

A prediction of the multi-block copolymer model is that microphase separation must persist as long as H4K16Ac is present in chromatin. In fact, H4K16Ac foci were unperturbed by treatments like 2 M NaCl extraction, which disrupts chromatin; transcription inhibition by DRB (5,6-Dichlorobenzimidazole 1-β-D-ribofuranoside), which reduces RNA pol II transcription by 98%; and heat shock (1h 45°C), which releases RNA pol II from the DNA [26] (Additional file 1: Figure S1). The only way to disrupt these foci was by formamide treatment, which works as a solvent for the electrostatic self-assembled polymers (Additional file 2: Figure S2).

These experiments contradict the excluding volume model [12] and are in agreement with the multi-block microphase separation hypothesis proposed in this study.

The next question about the genes in a TF is where they come from. Several studies have shown that genes in cis and in trans are able to interact in the same TF [5-7]. However, the analysis of chromatin spreads showed that collinear genes are active in the same cell at the same time. This guarantees that several H4K16Ac tracks are in close proximity. Therefore, most of these collinear TUs would probably aggregate in the same microsphere, as occurs in similar situations with the multi-block copolymers [34]. The experimental evidence from chromosome configuration capture analysis suggests that local chromatin is the primary source of interaction for any genomic loci [8]. Nevertheless, we cannot exclude the possibility that some genes located further away in the same chromosome or in another chromosome can interact due to proximity or chromatin folding.

Finally, a remarkable feature of TFs is their constant size across species and differentiation stages [23]. According to the multi-block copolymer model for chromatin organisation, the way to change the size of H4K16Ac foci (and consequently TFs) is by increasing the number of active genes in a given region or by unrestricting the mobility of the active chromatin. The latter has been reported in experiments using plasmids that rendered larger TFs than the endogenous ones [35,36].

In summary, we present evidence of the relationship between epigenetic marks and the TF structure. Our model proposes that active chromatin self-organises in the nucleus due to the special physical properties of H4K16Ac modified chromatin. Therefore, our model implies that chromatin becomes activated (H4K16Ac modified) before joining a TF. This is conceptually very different from current transcription factory model, which proposes that genes are targeted to TFs to “enhance production by concentrating the relevant machines, resources, and expertise in one place” [37].

## Materials and methods

### Transcription in vivo and in vitro

For in vivo transcription, cells grown on coverslips were incubated in presence of 2.5 mM BrU for several min.

For in vitro transcription, cells grown on coverslips were treated as described [3].

### Chromatin spreading

Cells (10^3^ cells in 5 *μ*l) were spotted onto a 22 × 50 mm glass slide and 5 *μ*l of lyses buffer were added (Lyses buffer: 1% sarkosyl, 25 U/ml ribonuclease inhibitor, 10 mM EDTA, and 100 mM Tris–HCl (pH 7.4)). After 10 min at 20°C, the slide was tilted to allow the drop to run down. Samples were air-dried and fixed in 4% Paraformaldehyde for 10 min. Clusters were defined as two o more hyper acetylated tracks in less than 100 Kb. For quantification of clusters of hyper acetylated chromatin between 150 and 200 tracks of hyper acetylated chromatin were analysed.

### Immunofluorescence

After blocking for non-specific antibody binding, immunolabelling was carried out as described [3]. For detection of primary transcripts, we used mouse anti-IdU/BrdU (5 mg/ml; Caltag Laboratories, Burlingame, CA). For detection of H4K16ac we used antibodies raised in rabbit and mouse (Serotec, Kidlington, UK, Abcam). RNA pol II hyperphosphorylated in Ser 2 was detected with H5 antibody (Covance). Secondary antibodies donkey anti-mouse IgG or IgM tagged with Cy3 (1/200 dilution; Jackson ImmunoResearch, Bar Harbor, ME) and donkey anti-rabbit IgG tagged with Alexa 488 (1/200; prepared using a Molecular Probes kit, Inc., Eugene, OR). DNA staining was performed with 200 nM TOPRO-3 (Molecular Probes) for 5 min. Then coverslips were mounted on slides using Vectashield (Vector laboratories), and images were collected using a Radiance 2000 confocal microscope (Bio-Rad Laboratories, Hemel Hempstead, Herts, UK), Distances were measured using EasiVision software (Soft Imaging Systems GmbH, Münster, Germany) and data exported to Excel (Microsoft) for analysis.

The degree of spreading of the chromatin was measured by hybridising the spreads with a fragment of DNA of 47.26 Kb; the spreading was 3.9 + 0.2 Kb/*μ*m.

### Microarrays and sliding window analysis

Mouse FCDP mix cells were used. cRNA synthesis and hybridisation to oligonucleotide array were performed as described [29].

The sliding window analysis was performed by applying a window of 500 Kb over the chromosomes and moved at 5 Kb steps along a chromosome to know whether the genes contained in that window were more likely to be transcribed together than just by chance.

## Authors' contributions

AZC-H, RPN and JER performed some of the immunocytochemical experiments and acquired data. SS and TE, performed the transcriptomic and the statistical analyses. CI participated in the draft the manuscript. VJB was in charge of the DNA fish experiments & FJI conceived of the study, and participated in its design and coordination and helped to draft the manuscript. All authors read and approved the final manuscript.

## Supplementary Material

Additional file 1: Figure S1 Stability of H4 K16Ac foci. Resistance of H4 K16Ac foci to various treatments that disrupted transcription or chromatin structure. The aspect of H4 K16Ac foci did not change after DRB treatment (2h 150 *μ*M) or heat shock (Hs) for 1h at 45°C. Both treatments led to the release of RNA pol II from the genes. These foci were also resistant to NaCl extraction (cells permeabilised with 0.05% Triton X100 for 5 min in PBS at 4°C followed by 10 min extraction with 2M NaCl for 10 min). The images were pseudo-coloured for display. The bottom bar shows the scale of pseudo-colours used. Click here for file

Additional file 2: Figure S2 H4 K16Ac foci are disassembled by formamide. The resistance of H4 K16Ac foci to formamide treatment. Cells were incubated for 5 min in PBS with different concentrations of formamide (0, 25, 50 and 100%) then fixed with 4% paraformaldehyde and immunolabelled with H4K16Ac antibodies. **(a)** The H4K16Ac foci were disassembled by formamide treatment, as can be seen from the change in the staining pattern, which is more diffuse and less intense than the control. The images were pseudo-coloured for display. **(b)** The deconstruction of the foci was quantified by the change in the pixel intensity variation coefficient (SD/mean). This analysis was performed by measuring the mean intensity and the standard deviation (SD) of the H4K16Ac signal of the nuclear areas in at least 200 cells for each treatment. The images were pseudo-coloured for display. The bottom bar shows the scale of pseudo-colours used. Click here for file
